# Editorial: Pharmacogenomics and pharmacomicrobiomics in type 2 diabetes mellitus (T2DM)

**DOI:** 10.3389/fendo.2023.1287807

**Published:** 2023-09-18

**Authors:** Jian-Quan Luo, Yan Shu, Wei Zhang

**Affiliations:** ^1^ Department of Pharmacy, the Second Xiangya Hospital, Central South University, Changsha, China; ^2^ Institute of Clinical Pharmacy, Central South University, Changsha, China; ^3^ Department of Pharmaceutical Sciences, School of Pharmacy, University of Maryland, Baltimore, MD, United States; ^4^ Department of Clinical Pharmacology, Xiangya Hospital, Central South University, Changsha, China; ^5^ Institute of Clinical Pharmacology, Central South University, Hunan Key Laboratory of Pharmacogenetics, Changsha, China; ^6^ Engineering Research Center of Applied Technology of Pharmacogenomics, Ministry of Education, Changsha, China; ^7^ National Clinical Research Center for Geriatric Disorders, Changsha, China

**Keywords:** pharmacogenomics, pharmacomicrobiomics, polymorphisms, microbiome, type 2 diabetes mellitus, precision medicine

Type 2 Diabetes mellitus (T2DM) is a complex and multifactorial metabolic disorder, caused by an interplay of genetic variations, unhealthy lifestyles and environmental factors. For many years, pharmacogenomics contributed to a great step towards precision medicine (1). The evidences from pharmacogenomics have revolutionized our understanding of the role of genetic variations in the T2DM pathogenesis and therapeutic response to antidiabetic drugs. In recent years, pharmacomicrobiomics, an extension of pharmacogenomics, has also provided unique insights into personalized medicine by investigating the interaction between drugs, host and gut microbiota (2). Therefore, we organized this Research Topic that aimed to shed light on the recent progress in pharmacogenomics and the emerging fields of pharmacomicrobiomics for T2DM.

In a series of contributions, multiple review articles have focused on the pharmacomicrobiomics for T2DM. The imbalance of human gut microbiota and their metabolites are increasingly considered to play a critical role in the development of T2DM and treatment outcomes of antidiabetic drugs. The review article of Chu et al. summarized the differences in the composition of gut microbiota between patients with T2DM and healthy individuals. Importantly, it also provided the current evidence on pharmacomicrobiomics of Western Medicine (WM) and Traditional Chinese Medicine (TCM) in T2DM. The effects of both WM and TCM could increase the relative abundance of health promoting bacteria, such as Akkermansia muciniphila, Blautia, and Bifidobacterium adolescent. Additionally, TCM might complement the efficacy of WM through alteration of microbiota. This review article of Wu et al. summarized the interaction between gut microbiota and its metabolites, including short-chain fatty acids, lipopolysaccharide, bile acids, trimethylamine-N-oxide, tryptophan and indole derivatives, and their role in the pathogenesis of T2DM. In addition, they discussed the potential strategies for prevention and treatment of T2DM by modulating the gut microbiota and its metabolites. The approaches included the use of probiotics, prebiotics, synbiotics, fecal microbiota transplantation, dietary interventions, bacteriophages, microbiota-targeted drugs and postbiotics. The review from Jia et al. highlighted the microbe-drug-host interactions, in particular for the antidiabetic drugs including metformin, thiazolidinedione, α-glucosidase inhibitors, sodium-glucose cotransporter 2 inhibitors, glucagon-like peptide-1 receptor agonists, dipeptidyl peptidase-4 inhibitors and traditional Chinese medicine. They also discussed the value of pharmacomicrobiomics findings as innovative potential personalized treatments for T2DM.

Post-transplant diabetes mellitus (PTDM) is a common and deleterious co-morbidity after solid organ transplantation. Intestinal dysbiosis may play a key role in the pathophysiology of drug-induced hyperglycaemia and diabetes mellitus (3). Based on complementary and coherent scientific evidence, the perspective article of Faucher et al. discussed the potential association between intestinal dysbiosis and PTDM, and provided arguments for the value of monitoring the microbiota diversity and function in solid organ transplantation.

Impaired glucose tolerance (IGT) is a necessary process for developing T2DM and an important stage where T2DM can be controlled and reversed. Thus, effective interventions are urgently needed (4). The original research of Guo et al. investigated the potential therapeutic targets of liraglutide in treatment of streptozotocin-induced impaired glucose tolerance (IGT) rats. Based on the tandem mass tag technique, the results revealed the target proteins of liraglutide, such as TBC1D13, PPIF, MPRIP, ME2, CYP51, DAD1, PTPA, TXNL1, ATG2B, BCL-2, etc. in the treatment of IGT. The clinical trial conducted by Yan et al. assessed the effect of early probiotic intervention in preventing conversion of patients with IGT to T2DM in the Probiotics Prevention Diabetes Program trial with follow up for 6 years. The patients with IGT were randomly assigned to either placebo treatment or probiotic supplementation with Bifidobacterium, Lactobacillus acidophilus and Enterococcus faecalis. After 6 years follow up, although active probiotic supplementation was safe, no significant difference was found in the cumulative incidence of developing T2DM (59.1% with probiotic treatment versus 54.5% with placebo).

Significant strides in the management of diabetic kidney disease (DKD) have evolved in parallel with the growing knowledge about its pathophysiological mechanisms (5). The original research of Wu et al. investigated the effects of dapagliflozin on DKD and gut microbiota composition during the progression of diabetes, performed 16S rRNA gene sequencing on fecal samples from C57BL/6 mice administrated with physiological saline, db/db mice administrated with physiological saline, and db/db mice treated with dapagliflozin at three timepoints of 14 weeks,18 weeks and 22 weeks. Based on their results, the authors highlighted the dynamic improvement of the gut microbiota over time accounting for the protective effect of dapagliflozin on DKD.

With regarding to the recent progress in pharmacogenomics T2DM, the Research Topic comprises two articles. The original research conducted by Song et al. evaluated the potential impact of two *PPARD* genetic variants (rs2016520 and rs3777744) on the therapeutic responses to exenatide in treating Chinese patients with T2DM.The study showed patients with *PPARD* rs2016520 TT genotype or rs3777744 G allele may have a poor response to exenatide therapy. One interesting point in this study is that *PPARD* rs2016520 and rs3777744 showed dramatically different allele frequencies in different ethnic populations. So, future studies are needed to explore the effects of *PPARD* rs2016520 and rs3777744 in the therapeutic responses to exenatide among other racial populations (6). The interactions of gene-lifestyle or gene-environment on the risk of T2DM is another important topic worthy of intensive investigation. Based on the data from the Korean Genome and Epidemiology Study Cohort, the original research article of Apio et al. investigated the association between dietary patterns and T2DM and conducted a gene-diet interaction analysis related to T2DM. The results showed that dietary pattern of poor amounts of antioxidant nutrients conferred to the risk of T2DM. The gene-diet interaction analysis indicated that dietary patterns affected pathway mechanisms in the development of T2DM.

In summary, the published articles in this Research Topic covered interesting findings and various aspects of the Research Topic, providing significant insights into the fields of pharmacogenomics and pharmacomicrobiomics in T2DM. In the future, the integration of pharmacogenomics and pharmacomicrobiomics will hold great promise for advancing personalized medicine of T2DM.

## Author contributions

JL: Conceptualization, Writing – original draft, Writing – review & editing, Project administration, Supervision. YS: Conceptualization, Supervision, Writing – review & editing, Project administration. WZ: Conceptualization, Project administration, Supervision, Writing – review & editing.

## References

[1] Pirmohamed M. Pharmacogenomics: current status and future perspectives. Nat Rev Genet (2023) 24(6):350–62. doi: 10.1038/s41576-022-00572-8 36707729

[2] Chen HQ Gong JY Xing K Liu MZ Ren H Luo JQ. Pharmacomicrobiomics: exploiting the drug-microbiota interactions in antihypertensive treatment. Front Med (Lausanne) (2021) 8:742394. doi: 10.3389/fmed.2021.742394 35127738 PMC8808336

[3] Luo JQ Ren H Chen MY Zhao Q Yang N Liu Q. Hydrochlorothiazide-induced glucose metabolism disorder is mediated by the gut microbiota via Lps-Tlr4-related macrophage polarization. iScience (2023) 26(7):107130. doi: 10.1016/j.isci.2023.107130 37456847 PMC10338205

[4] Rooney MR Fang M Ogurtsova K Ozkan B Echouffo-Tcheugui JB Boyko EJ. Global prevalence of prediabetes. Diabetes Care (2023) 46(7):1388–94. doi: 10.2337/dc22-2376 PMC10442190 37196350

[5] Naaman SC Bakris GL. Diabetic nephropathy: update on pillars of therapy slowing progression. Diabetes Care (2023) 46(9):1574–86. doi: 10.2337/dci23-0030 PMC10547606 37625003

[6] Luo JQ Ren H Liu MZ Fang PF Xiang DX. European versus Asian differences for the associations between paraoxonase-1 genetic polymorphisms and susceptibility to type 2 diabetes mellitus. J Cell Mol Med (2018) 22(3):1720–32. doi: 10.1111/jcmm.13453 PMC5824408 29314660

